# Occurrence of Carbapenem Resistance Producing Uropathogens Isolated From Refugees at the Nakivale Settlement in Isingiro District, Uganda: A Cross-Sectional Study

**DOI:** 10.7759/cureus.103254

**Published:** 2026-02-09

**Authors:** Lucas Ampaire, Michael Kabera, Calvin Cherop, Wilson Galimaka, Charles Nkubi Bagenda, Jazira Tumusiime, Byaruhanga Aggrey, Benson Okongo

**Affiliations:** 1 Department of Medical Laboratory Science, Mbarara University of Science and Technology, Mbarara, UGA; 2 Department of Medical Laboratory Sciences, Uganda Institute of Allied Health and Management Sciences, Kampala, UGA; 3 Department of Community Health, Mbarara University of Science and Technology, Mbarara, UGA; 4 Department of Epidemiology, Ministry of Health, Kampala, UGA

**Keywords:** antimicrobial resistance, carbapenem, community-acquired infections, urinary tract infections, uropathogens

## Abstract

Introduction

Carbapenem-resistant uropathogens are an emerging public health problem in sub-Saharan Africa. Such resistant pathogens are transferred easily from one community to another, given the rapid spread of mobile genetic elements containing carbapenemase genes. Uganda hosts refugees from several neighboring East African countries, many of whom are settled in the Nakivale Refugee Settlement. In our study, we analyze the extent of carbapenem-resistant uropathogens among refugees in one of the largest camps in Uganda.

Methods

We recruited 308 consenting participants. Each participant was tested for bacteriological urinary tract infection (bUTI) using standard urine cultures. Significant bacteriuria was determined as growth ≥10^5 ^CFU/mL and conventional biochemical tests were used for the identification of uropathogens. Phenotypic screening for carbapenem resistance was achieved using the modified Hodges test. End-point polymerase chain reaction (PCR) using 1.5% agarose gel in electrophoresis was used for the detection of carbapenemase genes. Bivariate and multivariate logistic regression using Stata, version 14.0, was done to identify the factors associated with carbapenem-resistant uropathogens. Ethical clearance was obtained from the Institutional Review Board of the Mbarara University of Science and Technology (MUST).

Results

The overall significant (≥10^5 ^CFU/mL) single bacterial growth was 29.0% (89/308). We detected phenotypic resistance in 55 (61.8%) out of 89 isolates and carbapenem resistance gene detected in 20 (22.5%) out of 89 isolates. *Escherichia coli *was the most common uropathogen, detected in 32 (36%) out of 89 isolates*. *The most frequently detected carbapenemase gene was *KPC *in 11 (55%) out of 20 isolates. The factors associated with carbapenem resistance were history of self-medication (odds ratio (OR)=5.09, 95% CI: 1.04-24.77, p=0.044), antibiotic use before laboratory diagnosis (OR=6.07, 95% CI: 1.77-20.81, p=0.004), and having spent more than five months on antibiotics (OR=8.52, 95% CI: 1.47-49.36, p=0.017).

Conclusion

The prevalence of carbapenem-resistant uropathogens isolated from refugees at Nakivale settlement was high. Accurate antimicrobial stewardship program implementation in refugee settlements is urgently needed. Screening and identification of carbapenem-resistant Enterobacterial careers among refugees at entry point could be helpful in mitigating the spread in refugee settlement.

## Introduction

Carbapenems are a class of antibiotic medicines used to treat infections caused by multidrug-resistant pathogens [1]. The emergence of widespread carbapenem resistance has become a significant public health concern [2]. Globally, antimicrobial resistance (AMR) is rapidly increasing, leading to outbreaks and treatment failures for community-acquired and nosocomial infections such as urinary tract infections (UTIs) caused by carbapenem-resistant Enterobacteriaceae [3].

In Uganda, UTIs remain the commonest community-acquired bacterial infection, with a prevalence of 25.29% [4]. These infections contribute to 16.3% of hospitalizations in all age groups among refugees and asylum seekers in developed countries [5]. Recent AMR situational analysis in Uganda reported an emergence of carbapenem resistance and a varying prevalence of nosocomial and community-acquired UTIs associated with carbapenem resistance estimated at 4%-30% [6,7]. However, this data is largely aggregated from different regional referral laboratories and hence may not accurately represent the refugee population that is served by lower health facilities where microbiology services are limited. Yet, empiric therapy is commonly practiced in the management of UTIs in refugee settlements without urine culture and antibiotic susceptibility testing [8]. Refugee host nations like Lebanon have indicated alarming evidence of an increase of carbapenemase resistance determining genes like OXA-48 and New Delhi Metallo beta-lactamase genes attributed to refugee crisis, antibiotic misuse, and water contamination in refugee camps [9]. Since inappropriate antibiotic therapy is associated with an increased risk of death from resistant pathogens, understanding local epidemiology is key to improving survival. However, a significant knowledge gap exists regarding etiology and carbapenem resistance among refugees in Uganda.

Carbapenems are the last choice antibiotics for most multidrug-resistant (MDR) organisms and the global trend of carbapenem resistance is exponentially rising due to the production of β-lactamases and carbapenemase that rapidly hydrolyzed β-lactam antibiotics of third and fourth generation and carried plasmid-encoded genes no longer susceptible to most of the antibiotic classes for treatment of UTIs, which cause superbug infections [10,11], especially in vulnerable communities like refugees.

To combat antimicrobial resistance, the Ministry of Health of Uganda developed a national action plan guided by the principles of the World Health Organization African Region (WHO-Africa) of early detection and diagnosis, proper prescriptions, and hand hygiene. However, these are not routinely followed in many of the local health setups, including Nakivale refugee camp [7,12,13].

Diagnosis of UTIs is routinely by use of urine dipstick for raised white blood cell count, esterases, and nitrites while urine culture and molecular detection of carbapenemase and β-lactamase-resistant genes in Uganda is limited by fewer capable laboratories [14]. Rudimentary diagnosis of carbapenem-resistant uropathogens results in increased mortality due to treatment failures, recurrence of infections, and complicated UTIs among refugees and the general public [15].

Uganda is a host for refugees from the Great Lakes region for over two decades. The continuous influx of unscreened refugees adds more burden to already struggling health care in Uganda due to a lack of or limited laboratory diagnostic services for detecting carbapenem-resistant uropathogens and general antibiotic resistance in refugee settlements like Nakivale [16].

Therefore, we focused on determining the prevalence and factors associated with carbapenem resistance determining genes isolated among uropathogens from refugees at Nakivale settlement, Isingiro district.

This article was previously posted to the ResearchSquare preprint server on August 17, 2024 (https://doi.org/10.21203/rs.3.rs-4658283/v1). 

## Materials and methods

Study design, population, settings, and ethical considerations

This cross-sectional study was carried out among refugees living in the Nakivale refugee settlement from May to July 2023. The settlement hosts over 119,587 refugees from the Democratic Republic of Congo, Burundi, Somalia, Rwanda, Ethiopia, and Eritrea. The Nakivale refugee settlement is officially recognized in 1960 through the Uganda Gazette General Notice No. 19 and is Uganda’s oldest and Africa’s largest refugee settlement covering over 180 square kilometers of land. It is the eighth largest settlement in the world, located in South-Western Uganda's Isingiro district, approximately 200 km from the capital Kampala. It is composed of three administrative zones - Base Camp, Juru, and Rubondo - with 74 villages, each hosting between 800 and 1000 people [17]. The settlement is served by one health center offering mainly outpatient services and with a basic laboratory. The health facility is headed by a clinical officer with two registered nurses and one laboratory technician. Patients that require further management are referred to another distant health facility about 50 km. Refugees are given a small piece of land for subsistence farming through the refugee self-reliance program (RSR) [18].

The study obtained clearance (MUST-2022-466, May 25, 2022) from the Institutional Review Board of the Mbarara University of Science and Technology before participants were involved in the study. Additionally, we obtained permission from the Office of the Prime Minister and the authorities managing the Nakivale refugee camp. Participation was voluntary, and each participant consented before enrollment into the study. Confidentiality was maintained throughout the study procedures, and all participants who had a laboratory-confirmed urinary tract infection were referred to the attending physician for appropriate management.

Sampling methods

We used a simple random sampling method to enroll study participants. The study included refugees who were clinically suspected to be having urinary tract infection based on signs and symptoms such as a burning sensation, a strong urge to urinate, cloudy urine, or bright pink-colored urine. Participants were excluded if they were treated at another facility for over 24 hours before screening, having a confirmed urinary tract infection at enrollment, or receiving anti-tuberculosis therapy.

Sample collection, handling, transportation, and storage

Participants were instructed to to wash hands, clean the urethral area, begin voiding, discard the first portion of urine, and collect approximately 20 mL of midstream urine into a wide-mouthed sterile container, avoid touching the inside of the container, and seal the container immediately. The samples were transported within two hours in a cool box, maintained at -4°C using ice packs, to the Microbiology Laboratory of the Mbarara Regional Referral Hospital. In case of any further delay, samples were preserved by refrigeration at 2-8°C for no more than 24 hours.

Sample processing for isolation of uropathogens

Each well-mixed urine was tested for uropathogen through the conventional microbiological method of urine culture. We used a quantitative technique with a loop of volume capacity 0.02 mL on cysteine lactose electrolyte deficient (CLED) agar that was aerobically incubated for 24-72 hours at 37°C. Additionally, isolates were Gram-stained and identified using conventional biochemical tests including growth patterns on triple sugar iron agar, Citrate utilization, indole production, Methyl Red, Vogues Proskauer, and motility. An active bacterial urinary tract infection was defined as any bacteria detected on culture in which a significant growth expressed equivalent to ≥10^5^ colony-forming units (CFU) per mL.

Phenotypic detection of carbapenem-resistant uropathogens

*Modified Hodge Test* (*MHT*)

We performed the modified Hodge test (MHT) as previously described using Muller Hinton agar (MHA; Oxoid, Basingstoke, UK). We prepared a standard suspension of a carbapenemase-producing reference strain-positive control (*Klebsiella pneumoniae* BAA 1706), and the test organism equivalent to 0.5 McFarland opacity in 5 mL of sterile saline. We spread a lawn of 1:10 dilution of *K. pneumoniae*, BAA 1706 on the surface of the MHA plate and allowed it to dry for five minutes. We placed a meropenem disc (10 µg, Oxoid) in the center of the inoculated plate and streaked the test organism in a straight line from the disk to the edge of the plate. The setup was incubated for 18-24 hours at 37°C. A clover leaf appearance (indentation of *K. pneumoniae*, BAA 1706 growth) on the streak of the test organism was interpreted as carbapenemase-producing organisms.

Genotypic detection of carbapenemase genes

DNA Extraction

DNA extraction was performed using the boiling method. Briefly, a single bacterial test/control isolate was emulsified in 1,000 µL of Tris-EDTA (TE) buffer in a labeled 1.5 mL microcentrifuge tube. The suspension was vortexed for one minute and centrifuged at 12,000×g for five minutes. The supernatant was discarded, and the pellet was washed twice with TE buffer under the same vortexing and centrifugation conditions. After the final wash, 100 µL of TE buffer was added to the pellet and vortexed until a homogeneous suspension was obtained. The suspension was heated at 95°C for one hour to lyse the cells and then allowed to cool to room temperature. It was subsequently centrifuged at 12,000×g for five minutes, and 80 µL of the supernatant containing genomic DNA was transferred to a newly labeled 1.5 mL tube and stored at −20°C until use.

PCR Master Mix Preparation

To make a final reaction volume of 25.0 µL, 12.5 µL Hot Start Taq2x master mix (M0496S, New England Bio-labs, Ipswich, MA, USA) was added to 1.0 µL forward primer (10 µM, New England Bio-labs), 1.0 µL reverse primer (10 µM, New England Bio-labs) and 5.0 µL DNA template and 5.5 µL RNAase-Free-H_2_O making up to final reaction volume. The primer sets that were used are indicated in Table 1.

**Table 1:** Primer Sets

Primer	Forward sequence	Reverse sequence	Length (bp)	Manufacturer
bla KPC	5’-GCTCAGGCGCAA CTGTAAG-3’	5’-AGCACAGCGGCAG CAAGAAAG-3’	150	New England Bio-labs
bla VIM	5’-GATGGTGTTTGGT CGCATA-3’	5’-CGAATGCG CAGCACCAG-3’	390	New England Bio-labs
bla OXA-48	5’-TATATTGCATTAAG CAAGGG-3’	5’-CACACAAATA CGCGCTAACC-3’	281	New England Bio-labs

Amplification and Cycling Conditions

We used a Classic K960 Thermal Cycler (Biomed Tech Holdings Limited, Hong Kong) for amplification using the following cycling conditions; initial denaturation at 95°C for three minutes, second denaturation at 95°C for 30 seconds, annealing at (52°C for OXA-48, 54°C for VIM and 55°C for KPC) for 30 seconds and elongation at 72°C for one minute) and the final extension cycle of 72°C for five minutes for 40 cycles.

Amplicon Detection

DNA Amplicon was electrophoresed using 1.5% agarose gel, in 1x Tris-Borate EDTA buffer (TBE), 5 µL Safe View Classic^TM^ DNA stain (cat # G108), 6x loading dye (Thermo Scientific, Waltham, MA, USA, #R0611), and DNA ladder/marker 1kb (New England Bio-labs, #N3231L). Electrophoresis was run at 200 V and 80m A for one hour. Bands were visualized using the Gene-Flash Transilluminator (Biobase, Jinan, China), primer set, and bands of the test were compared against the 1 Kb pair ladders of the respective genes.

Data analysis

We used Stata Version 14 (StataCorp LLC, College Station, TX) for analysis. Continuous variables were summarized as means and standard deviation while frequencies and percentages were reported for categorical variables. Participants’ characteristics were compared using chi-square test.

We additionally performed multiple logistic regression to determine which factors predicted a carbapenem-resistant uropathogen. All variables with a p<0.05 at univariate analysis and clinically significant were included in the multivariate logistic regression model. Crude and adjusted odds ratios with their corresponding 95% confidence interval were reported. Variables with a p value less than 0.05 were considered statistically significantly associated with carbapenem resistance-determining genes.

## Results

Baseline characteristics of the participants

The majority of the participants, 141 (54.8%) out of 308, were aged 20-29 years, and 175 (56.8%) were women. Over 50% of the participants came from the Democratic Republic of Congo, had formal education, were Catholic, and had at least one child (Table 2).

**Table 2:** Socio-demographic Characteristics of the Participants

Variable	Frequencies (n)	Percentages (%)
Age		
<20	25	8.1
20-29	138	44.8
30-39	82	26.6
40-49	41	13.3
50-59	15	4.9
60 and above	7	2.4
Gender		
Male	133	43.2
Female	175	56.8
Nationality		
Democratic Republic of Congo	169	54.9
Rwanda	92	29.9
Burundi	37	12.0
Ethiopia	2	1.6
Others	5	1.6
Religion		
Catholics	164	53.3
Protestants	61	19.8
Islam	67	21.8
Others	16	5.2
Education		
University	3	1.0
Vocational training	14	4.6
Secondary	129	41.9
No school	162	52.0
Household size		
Stay alone	11	3.6
No children	8	2.6
Less than one child	60	19.5
More than one child	229	74.4

Behavioral characteristics of the participants

The majority of the participants reported practicing self-medication (197, 64.0%), did not take all medication (203, 65.9%), few followed prescriptions (92, 29.9%), did not honor appointment dates (246, 79.9%), stopped medication (169, 55.0), few shared medicines (142, 46.1%), took unprescribed antibiotics (216, 70.1) and did not take antibiotics before laboratory diagnosis (172, 55.8%) (Table 3).

**Table 3:** Behavioral Characteristics of the Participants

Variable	Frequency (n)	Percentage (%)
Self-medication		
No	111	36.0
Yes	197	64.0
Take all medication		
No	203	65.9
Yes	105	34.1
Follow prescription		
No	216	70.1
Yes	92	29.9
Appointment keeping		
No	246	79.9
Yes	62	20.1
Stop medication		
Yes	169	54.9
No	139	45.1
Share medication		
No	166	53.9
Yes	142	46.1
Antibiotic use before laboratory diagnosis		
Yes	136	44.2
No	172	55.8

Medical characteristics of the participants

The majority of the participants had not been hospitalized (265, 86.0%), did not use invasive devices (171, 55.5%), the commonest comorbidity was tuberculosis (11, 3.6%), only seven (5.2%) had major surgery, and most took antibiotics for less than 30 days (188, 62.8%) (Table 4).

**Table 4:** Medical Characteristics of the Participants

Variable	Frequency (n)	Percentage (%)
Hospitalization		
No	265	86.0
Yes	43	14.0
Invasive device		
No	171	55.5
Yes	137	44.5
Surgical procedures		
No	286	92.9
Yes	22	7.1
Type of surgery		
No surgery	273	88.6
Major	12	3.9
Minor	23	7.5
Duration of antibiotics use		
<30 days	118	62.8
2-4 months	61	32.5
> 5 months	9	4.8

Distribution of uropathogens among the participants

**Figure 1:**
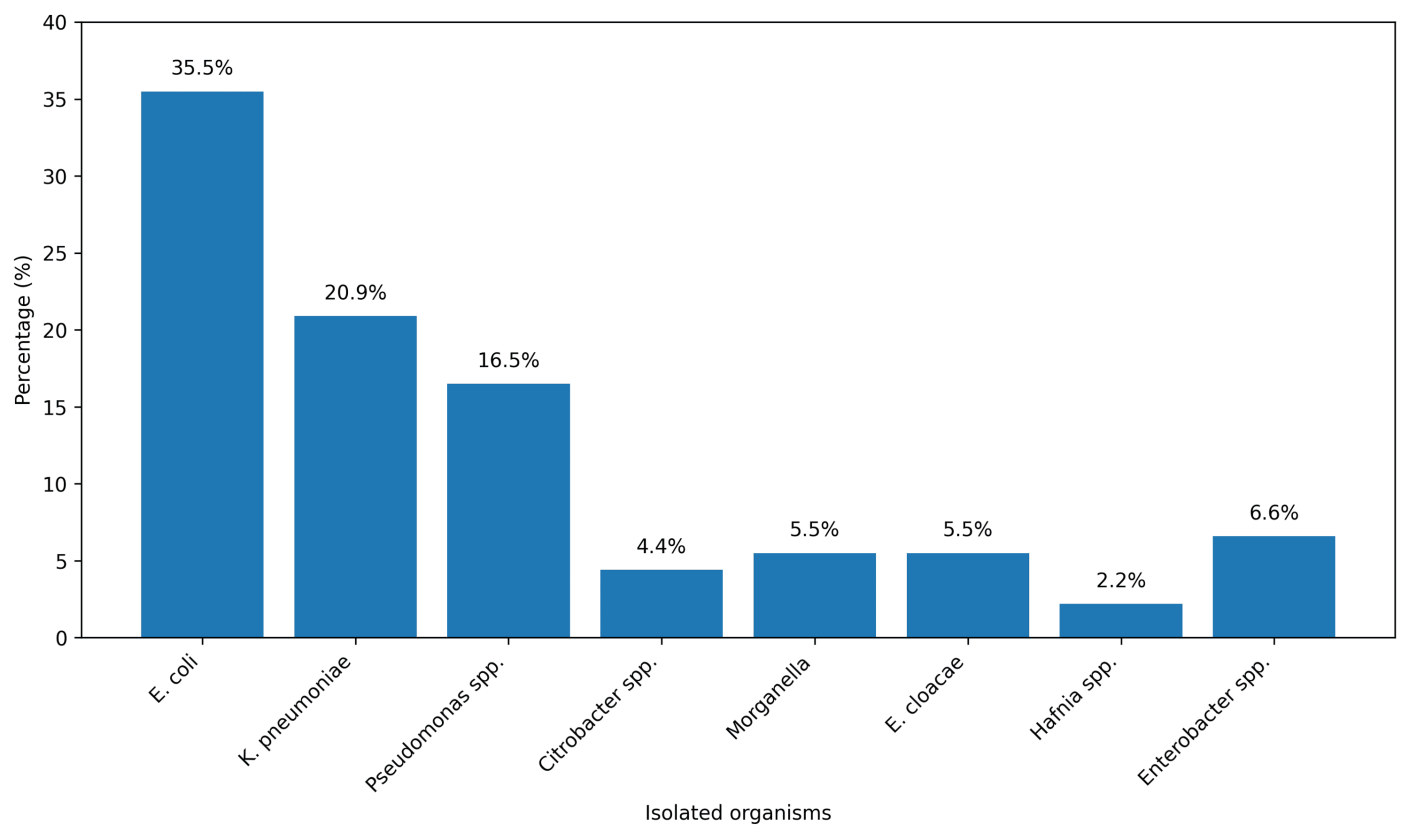
A bar graph showing distribution of isolates among partcipants

The most common uropathogens were *Escherichia coli* (32, 36.0%), and the least was *Hafinia *sp. (2, 2.0%) (Figure 1).

Phenotypic prevalence of carbapenem resistance

**Figure 2:**
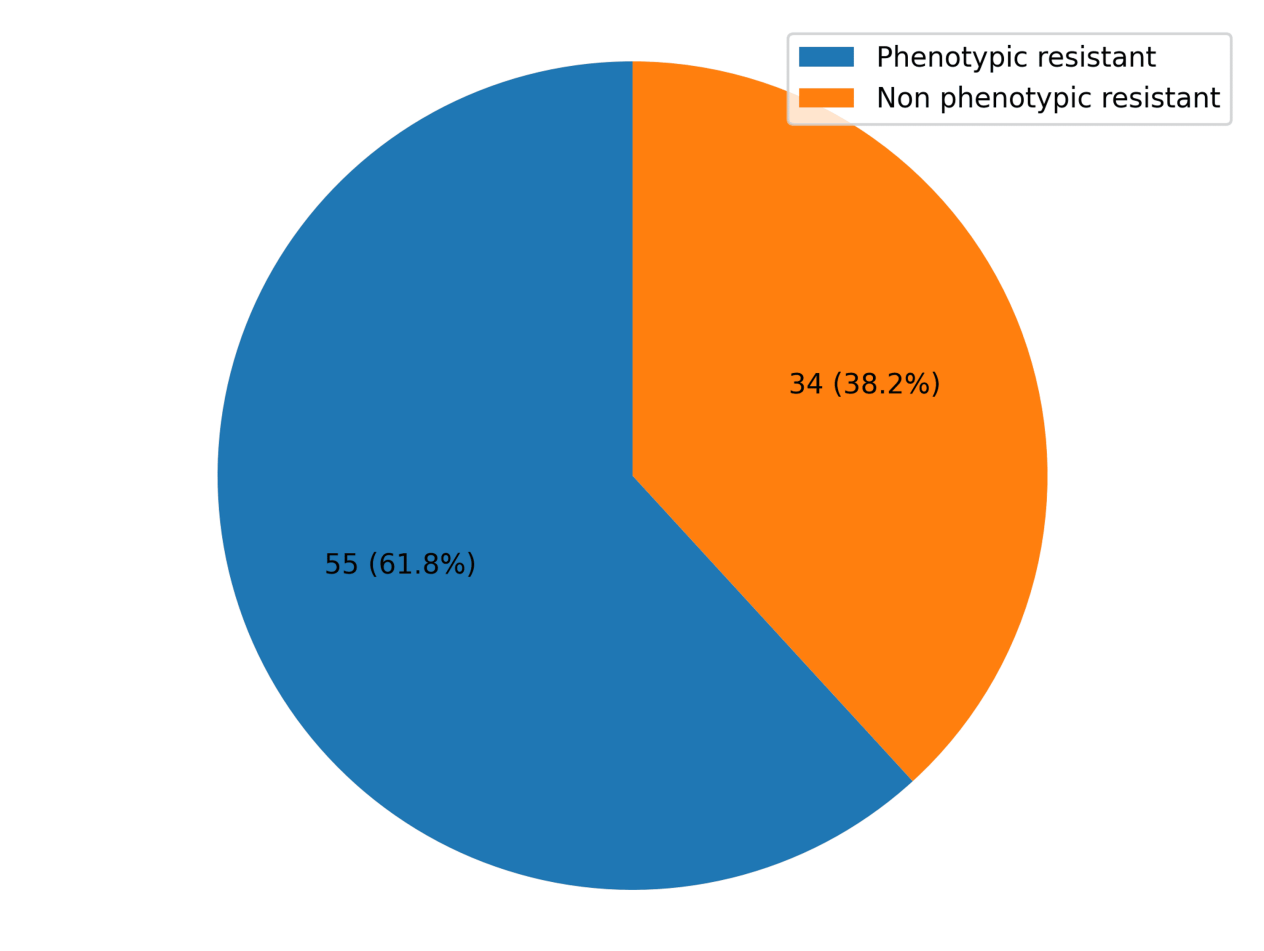
A pie-chart showing phenotypic prevalence of carbapenem resistance

Of the 89 uropathogens, 55 (61.8%) expressed phenotypic carbapenem resistance (Figure 2).

Genotypic prevalence of carbapenem resistance

The genotypic prevalence of carbapenem resistance was 22.5% (20/89) (Table 5) and the commonest gene harbored was bla-KPC (55%, 11/20) (Table 6).

**Table 5:** Genotypic Prevalence of Carbapenem Resistance

Carbapenemase gene occurrence	Frequency	Percentage
Present	20	22.5
Absent	69	77.5
Total	89	100.0

**Table 6:** The Commonest Carbapenemase Gene

Carbapenemase Gene	Frequency	Percentage
KPC	11	55
VIM	1	5
OXA-48	8	40
	20	100

A bivariate analysis of socio-demographic factors associated with carbapenem-resistant determining genes among uropathogens isolated from the refugees at the Nakivale settlement, Isingiro district, was done. Social demographic variables such as age, gender, nationality, religion, education, and household size had no statistically significant relationship with carbapenem resistance (p>0.05) (Table 7).

**Table 7:** Bivariate Analysis of the Social Demographic Factors Associated with Carbapenem Resistance cOR: Crude odds ratio; CI: confidence interval.

Variable	Carbapenem (CP) resistance		cOR (95%CI)	P value
	Non-CP resistant, n (%)	CP resistant, n (%)		
Age				
<20	24 (8.3)	1 (5.0)	1.00	
20-29	130 (45.1)	8 (40.0)	1.47 (0.17-12.35)	0.719
30-39	75 (26.0)	7 (35.0)	2.24 (0.26-19.13)	0.461
40-49	40 (13.9)	1 (5.0)	0.6 (0.035-10.04)	0.722
50-59	13 (4.5)	2 (10.0)	3.69 (0.30-44.69)	0.305
60 and above	6 (2.1)	1 (5.0)	4.00 (0.21-73.61)	0.351
Gender				
Male	126 (43.8)	7 (35.0)	1.00	
Female	162 (56.3)	13 (65.0)	1.44 (0.55-3.727)	0.447
Nationality				
Democratic Republic of Congo	158 (54.9)	11 (55.0)	1.00	
Rwanda	84 (29.2)	8 (40.0)	1.36 (0.52-3.53)	0.517
Burundi	36 (12.5)	1 (5.0)	0.39 (0.04-3.19)	0.386
Ethiopia	5 (1.7)	0 (0.0)	1	
Others	5 (1.7)	0 (0.0)	1	
Religion				
Catholics	154 (53.5)	10 (50.0)	1.00	
Protestants	58 (20.1)	3 (15.0)	0.79 (0.21-2.99)	0.737
Islam	61 (21.2)	6 (30.0)	1.51 (0.52-4.34)	0.440
Others	15 (5.2)	1 (5.0)	1.02 (0.12-8.57)	0.981
Education				
University	2 (0.7)	1 (5.0)	1.00	
Vocational training	13 (4.5)	1 (5.0)	0.15 (0.006-3.57)	0.244
Secondary	121 (42.0)	8 (40.0)	0.13 (0.01-1.61)	0.113
No school	152 (52.8)	10 (50.0)	0.13 (0.01-1.577)	0.110
Household size				
Stay alone	9 (3.1)	2 (10.0)	1.00	
No children	6 (2.1)	2 (10.0)	1.50 (0.16-13.74)	0.720
Less than one child	55 (19.1)	5 (25.0)	0.40 (0.06-2.43)	0.326
More than one child	218 (75.7)	11 (55.0)	0.22 (0.043-1.179)	0.078

A bivariate analysis of behavioral factors associated with carbapenem-resistant determining genes among uropathogens isolated from refugees at the Nakivale settlement, Isingiro district, was also done. The odds of carbapenem resistance were higher among participants who were practicing self-medication (crude odds ratio (cOR)=5.48, 95% confidence interval (CI)=1.24-24.07, p=0.024), sharing medication (cOR=2.91, 95% CI=1.09-7.80, p=0.033), and antibiotic use before laboratory diagnosis (cOR=4.14, 95% CI=1.46-11.70, p=0.007) (Table 8).

**Table 8:** Bivariate Analysis of the Behavioral Factors Associated with Carbapenem Resistance cOR: Crude odds ratio; CI: confidence interval.

Variable	Carbapenem (CP) resistance		cOR (95%CI)	P-value
	Non-CP, n (%)	CP, n (%)		
Self-medication				
No	109 (37.9)	2 (10.0)	1.00	
Yes	179 (62.2)	18 (90.0)	5.48 (1.24-24.07)	0.024
Take all medication				
No	187 (64.9)	16 (80.0)	1.00	
Yes	101 (35.1)	4 (20.0)	0.46 (0.15-1.42)	0.178
Follow prescription				
No	203 (70.5)	13 (65.0)	1.00	
Yes	85 (29.5)	7 (35.0)	1.28 (0.49-3.33)	0.605
Appointment keeping				
No	233 (80.9)	13 (65.0)	1.00	
Yes	55 (19.1)	7 (35.0)	2.28 (0.86-5.98)	0.094
Stop medication				
Yes	133 (46.2)	6 (30.0)	1.00	
No	155 (53.8)	14 (70.0)	0.49 (0.18-1.33)	0.167
Share medication				
No	160 (55.6)	6 (30.0)	1.00	
Yes	128 (44.4)	14 (70.0)	2.91 (1.09-7.80)	0.033
Antibiotic use before laboratory diagnosis				
Yes	121 (42.0)	15 (75.0)	1.00	
No	167 (58.0)	5 (25.0)	4.14 (1.46-11.70)	0.007

A bivariate analysis of medical factors associated with carbapenem-resistant determining genes among uropathogens isolated from the refugees at the Nakivale settlement, Isingiro district, was performed as well. The odds of carbapenem resistance are higher among participants whose duration of antibiotics use was five months or more (cOR=9.33, 95% CI=1.86-46.73, p=0.007). Variables such as hospitalization, invasive device, surgical procedures, type of surgery, and history of the use of antibiotics had no significant association with carbapenem resistance (p>0.05) (Table 9).

**Table 9:** Bivariate analysis of the medical factors associated with Carbapenem Resistance cOR: Crude odds ratio; CI: confidence interval.

Variable	Carbapenem (CP) resistance		cOR (95%CI)	P value
	Non-CP n (%)	CP n (%)		
Hospitalization				
No	249 (86.5)	16 (80.0)	1.00	
Yes	39 (13.5)	4 (20.0)	1.59 (0.50-5.02)	0.424
Invasive device				
No	160 (55.6)	11 (55.0)	1.00	
Yes	128 (44.4)	9 (45.0)	1.02 (0.411-2.54)	0.961
Surgical procedures				
No	269 (93.4)	17 (85.0)	1.00	
Yes	19 (6.6)	3 (15.0)	2.49 (6.672-9.28)	0.172
Type of surgery				
No surgery	258 (89.6)	15 (75.0)	1.00	
Major	10 (3.5)	2 (10.0)	3.44 (0.69-17.12)	0.131
Minor	20 (6.9)	3 (15.0)	2.58 (0.688-9.66)	0.159
Duration of antibiotics use				
<30 days	112 (65.5)	6 (35.3)	1.00	
2-4 months	53 (40.0)	8 (47.0)	2.81 (0.93-8.53)	0.067
>5 months	6 (3.5)	3 (17.7)	9.33 (1.86-46.73)	0.007

A multivariate analysis of the factors associated with carbapenem-resistant determining genes among uropathogens isolated from the refugees at Nakivale settlement, Isingiro district, was performed. Participants who had history of self-medication were 5.09 times more likely to experience carbapenem resistance as compared to those who were not self-medicating (OR=5.09, 95% CI: 1.04-24.77, p<0.05). Participants who practiced antibiotic use before laboratory diagnosis had 6.07 higher odds of developing carbapenem resistance as compared to those who didn’t (OR=6.07, 95% CI: 1.77-20.81, p=0.004). Participants who had spent more than five months on antibiotics were 8.52 times more likely to experience carbapenem resistance as compared to those who had spent less than one month (OR=8.52, 95% CI: 1.47-49.36, p=0.017) (Table 10).

**Table 10:** Multivariate Analysis of the Factors Associated with Carbapenem Resistance Genes CP: Carbapenem producers; cOR: crude odds ratio; aOR: adjusted odds ratio; CI: confidence interval. *Statistically significant at bivariate analysis. **Statistically significant at multivariate analysis.

Variable	CP, n (%)	cOR (95%CI)	P value	aOR (95%CI)	P-value
Duration of antibiotics use					
<30 days	6 (35.3)	Ref.		Ref	
2-4 months	8 (47.0)	2.81 (0.93-8.53)	0.067	3.10 (0.95-10.03)	0.059
> 5 months	3 (17.7)	9.33 (1.86-46.7)	0.007*	8.52 (1.47-49.36)	0.017**
Self-medication					
No	2 (10.0)	Ref.		Ref	
Yes	18 (90.0)	5.48 (1.24-24.07))	0.024*	5.09 (1.04-24.77)	0.044**
Shared medication					
No	6 (30.0)	Ref.		Ref.	
Yes	14 (70.0)	2.91 (1.09-7.80)	0.033*	1.73 (0.56-5.36)	0.340
Antibiotic use before diagnosis					
Yes	15 (75.0)	4.14 (1.46-11.70)	0.007*	6.07 (1.77-20.81)	0.004**
No	5 (25.0)	Ref.		Ref.	

## Discussion

In this study, the phenotypic prevalence of carbapenem resistance was 55 (61.8%). These findings were inconsistent with a study of carbapenem-resistant Enterobacteriaceae (CRE) done in Canada, and 13.0% of the prevalence was associated mainly with nosocomial infection because no patient reported seeking healthcare abroad or traveling to high carbapenem resistance endemic areas [19]. Our findings were consistent with 60% CRE reported in some European countries due to poor antibiotic prescription, which leads to multidrug resistance and complicated UTIs [20,21]. There is a continuous increase in CRE associated with complicated community and hospital-acquired infections in Third World countries, which calls for robust and effective antibiotic resistance control measures [22].

Our findings are consistent with reports from other East African countries where resistance rates were reported to be up to 70% in healthcare settings [23]. This could be because of similarities in healthcare and antibiotic stewardship challenges like limited access to routine culture and susceptibility testing, widespread self-medication, limited access to safe water, poor sanitation and unregulated antibiotic use [24,25] as reported in Africa. Additionally, Uganda, being a host to large refugee populations, faces a further strain on already constrained healthcare systems and increases the risk of inappropriate antibiotic use and spread of antimicrobial-resistant organisms. Other studies have also associated the varied spread of carbapenem resistance to genetic modification of Gram-negative Enterobacteriaceae [26].

Also, our findings were inconsistent with studies carried out in Italy (28.4%) and Spain (37%). These lower prevalences are due to implementation of antibiotic stewardship programs like screening migrants, continuous surveillance programs, well-equipped microbiology laboratories that offer culture and sensitivity, which were lacking at Nakivale refugee settlement [19]. A study done in the United States showed that 51.4% of CRE among patients suffering from UTIs was associated with empiric treatment, which was a common practice among the refugee community in the Nakivale settlement [27]. Conversely, lower prevalence of CRE was reported in Switzerland (0.3%), Israel (12%), and North Lebanon 1.7% [28] attributed to continuous surveillance and diagnostic services in Europe that were lacking at the Nakivale refugee camp.

In this study, the genotypic prevalence of carbapenem resistance was 20 (22.5%). This demonstrated that some of our participants in Nakivale were infected with bacteria that could have undergone plasmid Ampc ESBL transfer genes or porin mutations. However, our results were inconsistent with the findings in the study done on *K. pneumoniae* due to 43.1% capsular tying but similar to 22.8% genotypic prevalence of *E. coli* in tertiary hospitals in Uganda [29], although these studies focused on a single isolate and used multiplex polymerase chain reaction (PCR), which is more sensitive than gel electrophoresis.

Distribution of the resistant genes

The majority of genes detected were KPC 11 (55 %), OXA-48 8(40%) and the least was VIM 1 (5%). The distribution of these genes in our study was widely seen predominantly from *E. coli *and *K. pneumoniae* isolates. We did not do sequencing for these genes and therefore, we could not adequately describe this distribution.

A study done in the ICU unit at Imam Khomeini Hospital in Iran reported a 16.66% distribution of blaOxa-48. This was inconsistent with 40% distribution of that gene in our study, while blaVIM, blaKPC, and blaIMP were not detected. The difference could be explained by the fact that this study was done in a hospital setting in an ICU unit where infection control was being practiced unlike in Nakivale where people interacted randomly, possibly causing the spread of carbapenem-resistant uropathogens [30]. A clinical study conducted in Canada showed that CPE increased by 0.33/100,000 population despite most of the patients having no history of travel to high endemic areas implying that nosocomial transmission was the likely source of transmission of CRE and the distribution of carbapenemases was VIM (67%), KPC (55%), and New Delhi β-lactamase (22%), which was higher than findings of our study [19].

A study in the United Kingdom found that water reservoirs contaminated with IMP, KPC and VIM possessing Enterobacteriaceae around the hospital premises were major risk factors for CRE-causing nosocomial infections among terminally ill and immunocompromised patients [31]. Although we isolated CPO from urine only, refugees collected water from very few centralized water taps and boreholes, which are being shared with the local indigenous population, potentially increasing the risk of carbapenem transmission.

Variation in gene distributions like KPC (55%), OXA-48 (40%), and VIM (5%) was an indicator of crossover of genes due to mutations and possession of porin pores, which were the likely risk factors to multidrug and carbapenem resistance in the Nakivale refugee settlement [32]. The emergence of variant ST131 *E. coli* and blaOXA-244 caused carbapenem resistance [33] and virulent factors like motility and swarming among ESBL blaCTX-M-15, blaOXA-48-like genes and possession of integrons through mutations could help in the transfer and acquisition of carbapenemase-resistant genes [34] and VIM-1 [35]. However, we did not perform a virulent profiling of the isolates identified and we could not exactly determine which virulent factors were responsible for carbapenem resistance among refugees in the Nakivale refugee settlement.

In this study, participants who reported the use of antibiotics before laboratory diagnosis had 5.9 higher odds of carbapenem resistance compared to those with no history of use of antibiotics. Most of the participants reported taking medicines empirically to alleviate pain due to UTIs. This was accelerated by the lack of an advanced microbiology laboratory capable of culture analysis and sensitivity testing for a long time while in the camp and constantly falling ill with similar infections. Similarly, a study carried out in Indonesia and Japan found that 54.5% of patients who experienced carbapenem resistance were terminally ill and most of them reported prior use of antibiotics before diagnosis led to poor carbapenem resistance [36].

In this study, self-medication was associated with carbapenem resistance. This could be explained by delays in medical supplies to the Nakivale refugee camp that resulted in antibiotic selection pressure and a lack of strict regulatory measures in the Nakivale refugee settlement to combat carbapenem resistance. This phenomenon was reported in Saudi Arabia due to a lack of regulatory measures for taking prescription drugs [37]. Likewise, one study explained that people who self-medicated to alleviate infection symptoms ended up developing antibiotic resistance [38].

In this study, participants who had spent more than five months on antibiotics were 10 times more likely to have carbapenem resistance as compared to those who had spent less than one month. Similarly, a study in the United States reported that carbapenem resistance resulted from the prolonged use of antibiotics due to over-hospitalization [39]. These findings, although similar to some studies in hospital settings, may not be adequately comparable to a community-based refuge setting. However, the findings are from a single-center design, and the study used limited carbapenemase gene panels that may limit the generalizability of our findings. 

## Conclusions

The findings of this study demonstrate a high prevalence of phenotypic carbapenem resistance among refugees residing in Nakivale refugee settlement. Carbapenem resistance was strongly associated with a history of self-medication, prior antibiotic use, and prolonged antibiotic exposure exceeding five months, underscoring the role of inappropriate and extended antimicrobial use in driving resistance. *E. coli* and *K. pneumoniae* were identified as the predominant carbapenem-resistant isolates, reflecting their importance as key reservoirs of resistance in community and healthcare-associated infections. At the molecular level, the KPC carbapenemase gene was the most frequently detected, while VIM was the least expressed, suggesting variation in the distribution of resistance mechanisms within the settlement.

These findings emphasize the need to strengthen the diagnostic capacity of the laboratory for routine culture and antimicrobial susceptibility testing in Nakivale refugee settlement and other refugee camps in Uganda. Improving access to reliable diagnostic services, alongside promoting rational antibiotic use, is essential for early detection, appropriate treatment, and effective containment of carbapenem and other antimicrobial resistance in refugee and host communities across Uganda.
